# Understanding the endorsement of wife beating in Ghana: evidence of the 2014 Ghana demographic and health survey

**DOI:** 10.1186/s12905-020-00897-8

**Published:** 2020-02-11

**Authors:** Kwamena Sekyi Dickson, Edward Kwabena Ameyaw, Eugene Kofuor Maafo Darteh

**Affiliations:** 1 Department of Population and Health, University of Cape Coast, Cape Coast, Ghana; 2 The Australian Centre for Public and Population Health Research, Faculty of Health, University of Technology Sydney, Sydney, NSW Australia

**Keywords:** Endorsing, Wife beating, Ghana, Domestic violence, Intimate partner violence

## Abstract

**Background:**

Domestic violence (DV) has become a global burden. The high occurrence of intimate partner violence (IPV) across the globe has implications for the socioeconomic wellbeing and health of children and women.

**Methods:**

Data for the study was from the 2014 Ghana Demographic and Health Survey (GDHS). The association between approval of wife-beating and background characteristics of women was examined by the use of a Binary Logistic Regression model.

**Results:**

A higher proportion of respondents were from urban areas (53.7 and 52.2% women and men respectively). The ages of women ranged from 15 to 49 (mean = 30, SD = 9.7) whilst the age range of men was 15–59 (mean = 32, SD = 12.5). Twenty-four percent of the men and 23% of the women were within the richest wealth category. The results showed that few women (6.3%) and men (11.8%) had attained higher education. Both women (AOR = 1.3; CI = 1.01–1.24) and men (AOR = 2.2; CI = 1.72–2.76) aged 15–24 had higher odds of approving wife-beating than those aged 35–49 (reference category). Poorest women (AOR = 2.7; CI = 2.14–3.38) and men (AOR = 1.7; CI = 1.11–2.69) alike had higher odds of approving wife-beating, as compared with those in the richest wealth status (reference category). As compared to research participants with higher/tertiary education, both women (AOR = 5.1; CI = 3.52–7.51) and men (AOR = 4.2; CI = 2.37–7.16) without any formal education were found to be at higher odds to approve wife-beating; however, this observation seems to decline as one’s educational status advances.

**Conclusion:**

Age, wealth status, level of education, frequency of listening to radio, frequency of reading newspaper/magazine, frequency of watching television, ethnicity, and religion were found to be significantly associated with Ghanaian men and women’s approval of wife-beating. Policies, interventions, and campaigns must target Ghanaians without formal education and young adults on the need to uphold human rights in order to dissuade them from endorsing intimate partner violence. Mass media has also proven to be a protective factor against domestic violence approval and, as such, much progress can be made if utilised by human rights activists, especially through radio, magazine and television broadcasting.

## Background

Domestic violence (DV) has become a global burden [1, 2]. The WHO [2] reported that violence against women constitutes a major public health concern affecting a third of women globally [2]. The high occurrence of intimate partner violence (IPV) against women across the globe and the ensuing socioeconomic costs together with related health implications for women and their children have been documented [3, 4]. Domestic violence (DV) constitutes a pattern of coercive approaches including but not restricted to psychological, physical, social, emotional, sexual, and economic mishandling effected by one person against an intimate partner, usually motivated by the aim of creating and retaining power and or control [5]. Historically, five principal set of issues (male aggression, sociological factors, individual psychological factors, poverty and pervasiveness of beliefs of violence) [6, 7] have been advanced to explain the factors allied with intimate partner violence.

As acknowledged by the patriarchy theory, perpetration of domestic violence is reinforced by patriarchal ideologies. In patriarchal societies, males demonstrate superiority and gain control over females [8]. Patriarchy subordinates women and renders them more prone to violence in six main structures namely: patriarchal relations in paid work, patriarchal mode of production, male violence, patriarchal relations in the state, patriarchal relations in sexuality, and patriarchal relations within cultural institutions [9]. In Africa, violence is explained by two main ideologies: the first describing experiences of violence within the family and the second taking into account the changing definitions of domestic violence [10]. Sometimes, it is argued that both men and women experience domestic physical violence; however, women usually experience it most [11].

Studies have stressed individual psychology, male aggression, and interpersonal relationships within families as the dominant factors reinforcing DV [12–14]. Tactics for perpetrating DV include dominance, humiliation, isolation, threat, intimidation as well as denial and blame. The WHO [3] noted that Africa, South-Eastern Asia and the Eastern Mediterranean have the highest prevalence of physical domestic violence, with the least being recorded in Europe and the Western Pacific. Violence perpetration is significant among women in the childbearing age group (15–49 years)—a critical period for maternal health [15, 16]. Consequently, a couple of responses have been advanced to curtail this phenomenon, including the Declaration on the Elimination of Violence Against Women (DEVAW) and UN Special Rapporteur on Violence Against Women.

In Ghana, physical domestic violence manifests in several dimensions, including slapping or throwing things at someone, pushing, hitting, attacking with a weapon, choking, or strangling [16]. The most common forms of physical violence in Ghana over a lifetime include slaps or being hit with thrown objects, followed by being hit by another person [16]. However, varied implications emerge depending on the specific victim, his/her age, the intensity of the violence, and consistency of torment the person experiences [17]. Living under persistent threat, fear, and humiliation constitute some of the emotional states developed in the memories of victims [17].

Physical domestic violence has several implications for women. Victims are more prone to induced abortion, HIV, depression, and other ill health conditions such as anxieties, phobias, irritable bowel syndrome, and gynecological problems [3]. Women who experience physical domestic violence usually have babies with low birth weight [3]. These and other factors prompted the Ghana government to enact some laws to safeguard the rights and privileges of women and children. Among these are the 1992 Constitution, which prohibits sex-based discrimination, and criminalizes some harmful cultural practices such as widowhood rites. Additionally, the parliament of the Republic of Ghana enacted the Domestic Violence Act (Act 732). Beyond these, other efforts have been initiated over the past years to lessen the incidence of DV. For instance, the first legislative effort of the country acknowledged the intention of the global community in advocating women’s rights [18].

In a typical Ghanaian society, a woman’s inability to fulfill her gender roles is interpreted as “disobedience” [19]. Instances where a woman deserves to be beaten include denying her husband sex, and failure to execute her household duties [1, 19]. The Ghana Combined CEDAW Report noted that, to a greater extent, women are considered to be inferior, in need of protection, and are to conform to the orders of an authority, usually males, throughout their lifetime [1]. This phenomenon confers so much opportunity for men to chastise women and also consider beating as an effective tool for bringing women to order [1].

Most women are reluctant to report domestic violence due to its acceptance and the belief in women’s obligation to succumb to men [16]. In the light of this, domestic violence is considered “normal”, thereby hindering institutions from sanctioning culprits [19]. In some instances, women are considered to attract abuse by being independent (because it is assumed to threaten their husband’s superiority) and this worsens if the woman’s independence offers her the chance to augment her husband’s income [20]. Studies on IPV in Ghana have either focused on emotional violence [21] or are limited to the views of women alone [22–24]. These studies have not considered the main perpetrators of violence, men. As such, there is the need to fill this literature gap. This paper, therefore, contributes to the literature by examining the drivers deepening the endorsement of wife beating among both males and females in Ghana.

## Methods

### Data

The data for the study was obtained from both men’s and women’s files from the 2014 Ghana Demographic and Health Survey. The Demographic and Health Survey (DHS) captures data on various aspects of women’s health and wellbeing, including issues of physical domestic violence. The survey is a nationwide survey, with a representative sample of 9396 women and 4388 men aged 15*–*49 and 15*–*59 respectively. However, the actual samples for this study were 9387 and 4387 for women and men respectively due to non-response. The 2014 GDHS was conducted using an updated frame from the 2010 Population and Housing Census, which was prepared by the Ghana Statistical Service (GSS). The survey followed a two-stage sample design in order to allow estimates of core indicators at the national level. The first phase constituted a selection of sample points (clusters) involving enumeration areas (EAs) which were outlined for the 2010 PHC in which 427 clusters were designated constituting 216 from urban and 211 from rural areas. The second stage utilised systematic sampling of households, in which household inventory operation was conducted in all the identified EAs. Afterward, the households considered for the survey were selected from the list randomly [22].

### Definition of variables

Approval or justification of wife-beating was the outcome variable. Justification of a husband to beat the wife was asked under five conditions: if wife burns the food; if wife neglects the children; if wife argues with husband; if wife goes out without telling the husband; and if wife refuses to have sex with the husband. All these were asked as yes = 1 or no =0. An index was created with all the “yes” and “no” answers, with scores ranging from 0 to 5. The score 0 was labelled as “not approve” and 1 to 5 was labelled as “approve” [23, 24]. A dummy variable was generated with ‘0’ score being respondents who do not approve of wife-beating and ‘1’ being respondents who approve at least one of the five questions justifying wife-beating.

The explanatory variables were age, residential status, wealth, education, religion, and ethnicity. Inclusion of religion and ethnicity was as a result of inconsistencies in arguments about their influence on the approval of domestic violence in the literature [23, 25]. Additionally, some behavioural factors were considered and these are the frequency of listening to radio, frequency of reading newspaper/ magazine, and frequency of watching television. These behavioural factors were considered because campaigns against domestic violence in the country are communicated through these various channels. Also, some variables were recoded in order to ensure clarity of the results. With this, religion was recoded as Christian = 1; Islam = 2; Traditional/spiritual = 3; Other = 4. Age was also recoded into 15–24 = 1; 25–34 = 2; 35–49 = 3 and 50–59 = 4.

### Data analysis

Data analysis was carried out using STATA version 13. Since the outcome variable was dummy, binary logistic regressions were conducted. Two models were constructed in all; Model 1 and 2 report for females and males respectively. They looked at the combined effect of socio-demographic variables and behavioural factors on approval or justification for wife-beating. Survey weights were factored into both descriptive and inferential analyses in order to offset the challenges of under- and over-sampling which are usually associated with national surveys. Specifically, the binary logistic regression was employed, given that this technique is more appropriate for dichotomous variables. A key assumption underlying the binary logistic regression model is that the dependent variable should be dichotomous in nature and the data should not have any outlier.

## Results

### Descriptive results

More than half of the respondents reside in urban areas (53.7 and 52.2% women and men respectively) as indicated in Table 1. The ages of women ranged from 15 to 49 (mean = 30, SD = 9.7) whilst the age range of men was 15–59 (mean = 32, SD = 12.5). Significant proportions of both women (23.4%) and men (24.2%) were within the richest wealth category. The results showed that few women (6.3%) and men (11.8%) had attained higher education. As noted in the 2010 Population and Housing Census that Christianity is the leading religion in Ghana [26, 27], the study found that most women (80.1%) and men (72.5%) were Christians.

**Table 1:** Background characteristics of respondents

Explanatory Variable	Females (n = 9387)		Males (*n* = 4387)	
	%	N	%	N
*Residence*				
Urban	53.7	5043	52.1	2285
Rural	46.3	4344	47.9	2102
*Age*				
15–24	34.4	3233	32.9	1443
25–34	31.7	2973	26.0	1141
35–49	33.9	3181	29.3	1285
50–59	–	–	11.8	518
*Wealth status*				
Poorest	16.1	1512	17.1	751
Poorer	17.4	1636	17.8	779
Middle	20.6	1936	19.0	835
Richer	22.5	2116	21.9	960
Richest	23.4	2187	24.2	1062
*Level of Education*				
No education	19.1	1790	10.7	470
Primary	17.8	1670	13.5	590
Secondary	56.8	5331	64.0	2809
Higher/tertiary	6.3	596	11.8	518
*Religion*				
Christian	80.1	7524	72.5	3182
Islam	15.2	1421	17.6	771
Traditional/spiritual	2.0	191	3.7	164
Other	2.7	251	6.2	270
*Frequency of reading newspaper/ magazine*				
Not at all	81.1	7615	64.7	2837
Less than once a week	10.2	956	17.9	785
At least once a week	8.7	816	17.4	765
*Frequency of listening to radio*				
Not at all	15.6	1468	5.5	242
Less than once a week	32.1	3017	16.1	708
At least once a week	52.3	4902	78.4	3437
*Frequency of watching television*				
Not at all	23.5	2203	16.8	735
Less than once a week	25.7	2415	18.8	827
At least once a week	50.8	4769	64.4	2825
*Ethnicity*				
Akan	50.1	4701	49.0	2151
Ga/Dangme	7.7	727	9.0	393
Ewe	13.5	1265	13.6	595
Guan	2.3	216	2.0	87
Mole – Dagbani	14.8	1388	14.4	630
Grusi	2.9	269	2.5	112
Gurma	5.8	545	5.8	255
Mande	0.9	85	1.1	48
Other	2.0	191	2.6	116

Computed from 2014 Ghana Demographic and Health Survey

As indicated in Table 2, wife-beating is justified: if wife goes out without telling the husband (females 16.5% and males 6.6%); if wife neglects the children (females 21.0% and males 8.2%); if wife argues with husband (females 15.5% and males 5.9%); if wife refuses to have sex with husband (females 12.8% and male 4.8%); and if wife burns food (females 7.4% and males 2.8%).

**Table 2:** Justification of wife beating

Explanatory Variable	Females (*N* = 9387)		Males (N = 4387)	
	%	n	%	n
Wife beating justified if wife goes out without telling husband				
No	83.5	7838	93.4	4096
Yes	16.5	1549	6.6	288
Wife beating justified if wife neglects the children				
No	79.0	7419	91.8	4027
Yes	21.0	1968	8.2	359
Wife beating justified if wife argues with husband				
No	84.4	7919	94.1	4128
Yes	15.6	1468	5.9	258
Wife beating justified if wife refuses to have sex with husband				
No	87.2	8243	95.2	4177
Yes	12.8	1144	4.8	209
Wife beating justified if wife burns food				
No	92.6	8691	97.2	4265
Yes	7.4	696	2.8	121
At least one justification of wife beating				
No	71.7	6731	87.5	3838
Yes	28.3	2656	12.5	548

Computed from 2014 Ghana Demographic and Health Survey

### Multivariate logistic regression results

As illustrated in Table 3, Model 1 is a combination of socio-demographic and behavioural factors and approval of wife-beating among females. Model 2 also looked at how socio-demographic and behavioural factors predicted the approval of wife-beating among males. With regard to the socio-demographic characteristics, both women (AOR = 1.4; CI = 1.19–1.52) and men (AOR = 2.2; CI = 1.70–2.74) aged 15–24 had higher odds of approving wife-beating than those aged 35–49 (reference category). Women with poorest (AOR = 2.2; CI = 1.77–2.86) and poorer (AOR = 2.2; CI = 1.77–2.86) wealth statuses and men with richer wealth status (AOR = 1.5; CI = 1.01–2.20) had higher odds of approving wife-beating, as compared with those in the richest wealth status (reference category). As compared to research participants with higher/tertiary education, both women (AOR = 4.3; CI = 2.89–6.27) and men (AOR = 3.0; CI = 1.71–5.36) without any formal education were found to have the highest odds of approving wife-beating.

**Table 3:** Binary regression on approval of wife beating

Variables	Females	Males
	Model 1	Model 2
*Age*		
15–24	1.4***(1.19–1.52)	2.2***(1.70–2.74)
25–34	1.0(0.92–1.28)	1.4**(1.05–1.77)
35–49	1	1
50–59	–	1.0(0.68–1.32)
*Residence*		
Urban	1	1
Rural	1.0(0.90–1.16)	1.1(0.87–1.42)
*Wealth status*		
Poorest	2.2***(1.77–2.86)	1.3(0.84–2.12)
Poorer	2.2***(1.77–2.86)	1.4(0.90–2.11)
Middle	2.0***(1.63–2.40)	1.5*(1.01–2.20)
Richer	1.4***(1.16–1.69)	1.1(0.75–1.62)
Richest	1	1
*Education*		
No education	4.3***(2.89–6.27)	3.0***(1.71–5.36)
Primary	3.3***(2.23–4.82)	2.2**(1.28–3.92)
Secondary	2.7***(1.84–3.84)	2.1**(1.23–3.45)
Higher/tertiary	1	1
*Ethnicity*		
Akan	1	1
Ga/dangme	0.8*(0.61–0.98)	1.1(0.71–1.58)
Ewe	0.8*(0.69–0.96)	0.5***(0.31–0.66)
Guan	1.5**(1.13–1.97)	1.1(0.64–1.96)
Mole-dagbani	0.9(0.79–1.07)	1.1(0.80–1.41)
Grusi	1.0(0.78–1.24)	1.5(0.99–2.27)
Gurma	2.5***(2.04–3.09)	2.0***(1.45–2.84)
Mande	0.7(0.43–1.04)	0.7(0.31–1.76)
Other	0.9(0.64–1.32)	1.2(0.67–2.25)
*Religion*		
Christian	1	1
Islam	1.7***(1.44–1.91)	0.8(0.64–1.08)
Traditional/spiritual	1.1(0.83–1.50)	1.2(0.86–1.72)
Other	1.1(0.85–1.43)	1.6**(1.13–2.15)
*Frequency of listening to radio*		
Not at all	1	1
Less than once a week	0.7***(0.64–0.85)	1.3(0.64–1.08)
At least once a week	0.8***(0.69–0.89)	0.9(0.62–1.21)
*Frequency of reading newspaper/ magazine*		
Not at all	1	1
Less than once a week	1.0(0.79–1.15)	0.9(0.64–1.15)
At least once a week	0.6***(0.47–0.78)	0.6**(0.39–0.80)
*Frequency of watching television*		
Not at all	1	1
Less than once a week	0.9*(0.74–0.98)	0.9(0.70–1.18)
At least once a week	0.8**(0.71–0.92)	0.8*(0.59–0.98)

Computed from 2014 Ghana Demographic and Health Survey

*p* < 0.05* *p* < 0.01**p < 0.001***

When compared with Christians, women from Islam religion (AOR = 1.7; CI = 1.41–1.91) were more likely to approve wife-beating. A similar observation was made among men affiliated to other religious denominations (AOR = 1.6; CI = 1.13–2.15) (see Table 3). Women who listened to radio less than once a week (AOR = 0.7; CI = 0.64–0.85) had lesser odds of approving wife-beating, as compared with women who do not listen to radio at all. It was also realised that women who read newspapers/magazines at least once a week had less tendency of approving wife-beating (AOR = 0.6; CI = 0.47–0.78). Also, women who watched television at least once a week (AOR = 0.8; CI = 0.71–0.92) had lesser odds of approving wife-beating.

## Discussion

The study examined the approval of wife-beating in Ghana from the perspectives of women and men. This study is of much essence, considering the increasing reported rates of domestic physical violence in the country [28, 29]. Previous evidence suggests that accepting or being permissive toward violence contributes positively toward its perpetration [30]. Meanwhile, little has been documented about the perception of Ghanaians on approval of the phenomenon. Generally, no much variation existed among men and women in terms of their approval of wife-beating (age, wealth status, and education); however, inconsistent results were observed in some instances (such as religion).

This study has observed high inclination of both young men and women toward the approval of wife-beating in Ghana. Possible explanations might include their adherence to cultural tenets [31, 32]. However, the older ones may hold a contrary view due to their experiences about the implications of violence over the years which the younger ones may not be aware of. There has been an inconsistent argument in the literature concerning age and approval of domestic physical violence. Whilst the study contradicts findings that report a low tendency of approval among young adults [33, 34], it is consistent with evidence suggesting otherwise [35].

In terms of wealth, poor men and women approved wife-beating. Having a significant proportion of poor women to approve domestic physical violence might be as a result of their dependence on their husbands for livelihood [24, 36]. When someone depends on somebody for a livelihood, the dependant is more likely to accept and interpret almost all actions of the independent person as acceptable, even if the actions are detrimental to the wellbeing of the dependant [37]. Also, poor men might consider wife-beating as a strategy of easing the psychological trauma induced by their low wealth standing [38]. When men are consistently unable to contend the financial burden of their families, they can be frustrated and irritated at the least provocation and, as a result, such men might consider perpetration of physical violence as a strategy for easing the burden mounted by their economic situation [39, 40]. Some studies in sub-Saharan Africa have made a similar observation by noticing a substantial association between financial reliance and physical violence [23, 24, 34, 41, 42].

Education has been noted to subside the tendency of someone to approve wife-beating. Education promotes autonomy and liberation from suppression [23]. Similar findings have been revealed in other countries [25, 43, 44]. Again, an earlier study has also indicated that Ghanaians without any formal education, especially females, are more probable to support violent ideologies. This, therefore, suggests that formal education is essential if approval of violence perpetration can decline in Ghana.

The study revealed that women having exposure to mass media (radio, newspaper/magazine, and television) were less likely to approve wife-beating. However, men who listened to radio had a higher tendency to approve wife-beating, as compared to men who did not listen to radio. This shows that exposure to mass media (radio) has varied implications on perception about domestic violence approval among men and women. The finding is not surprising in light of the fact that most women stay home and interact with mass media all day whilst men leave for work. The long stay at home creates a conducive environment for women to enhance their depth of knowledge on the implications of physical violence because they tend to have much information from mass media than men.

## Conclusion

In conclusion, females justify wife-beating than males. Age, wealth status, level of education, frequency of listening to radio, frequency of reading newspaper/magazine, frequency of watching television, ethnicity, and religion were found to be significantly associated with Ghanaian men and women’s approval of wife-beating. Policies, interventions, and human right campaigns must target Ghanaians without primary educational status and young adults on the need to uphold human rights in order to dissuade them from endorsing intimate partner violence. Mass media has also proven to be a protective factor against domestic violence approval and, as such, much progress can be made if utilised by human rights activists, especially radio/magazine and television broadcasting. Again, rural Ghanaian adults must be targeted in order to achieve holistic improvement in the reduction of domestic physical violence, particularly wife-beating.

## Data Availability

The report and dataset are freely available to the public at www.measuredhs.com, upon request and submission of a consent paper.

## Abbreviations

CHRAJ: Commission for Human Rights and Administrative Justice
DEVAW: Declaration on the Elimination of Violence Against Women
DHS: Demographic and Health Survey
DOVVSU: Domestic Violence and Victim Support Unit
DV: Domestic Violence
IPV: Intimate Partner Violence
UN: United Nations
WHO: World Health Organization
